# 3-(4-Chloro­phenyl­sulfin­yl)-2,5,7-tri­methyl-1-benzofuran

**DOI:** 10.1107/S1600536810032228

**Published:** 2010-08-18

**Authors:** Hong Dae Choi, Pil Ja Seo, Byeng Wha Son, Uk Lee

**Affiliations:** a Department of Chemistry, Dongeui University, San 24 Kaya-dong Busanjin-gu, Busan 614-714, Republic of Korea; b Department of Chemistry, Pukyong National University, 599-1 Daeyeon 3-dong, Nam-gu, Busan 608-737, Republic of Korea

## Abstract

In the title compound, C_17_H_15_ClO_2_S, the O atom and the 4-chloro­phenyl group of the 4-chloro­phenyl­sulfinyl substituent are located on opposite sides of the plane through the benzofuran fragment; the 4-chloro­phenyl ring is approximately perpendicular to this plane [dihedral angle = 87.12 (3)°]. In the crystal structure, mol­ecules are linked through a weak inter­molecular C—H⋯O hydrogen bond, and by weak C—S⋯π [3.394 (2) Å] and C—Cl⋯π [3.800 (2) Å] inter­actions.

## Related literature

For the pharmacological activity of benzofuran compounds, see: Aslam *et al.* (2006 ▶); Galal *et al.* (2009 ▶); Khan *et al.* (2005 ▶). For natural products with benzofuran rings, see: Akgul & Anil (2003 ▶); Soekamto *et al.* (2003 ▶). For related structures, see: Choi *et al.* (2010*a* ▶,*b* ▶).
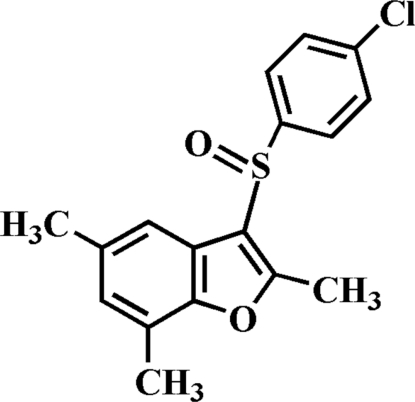


## Experimental

### 

#### Crystal data

C_17_H_15_ClO_2_S
*M* *_r_* = 318.80
Triclinic, 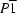

*a* = 6.0790 (12) Å
*b* = 10.2232 (19) Å
*c* = 12.514 (2) Å
α = 84.474 (9)°
β = 80.121 (9)°
γ = 85.991 (9)°
*V* = 761.5 (3) Å^3^
*Z* = 2
Mo *K*α radiation
μ = 0.39 mm^−1^
*T* = 173 K
0.33 × 0.29 × 0.29 mm

#### Data collection

Bruker SMART APEXII CCD diffractometer
Absorption correction: multi-scan (*SADABS*; Bruker, 2009 ▶) *T*_min_ = 0.883, *T*_max_ = 0.897
13813 measured reflections
3789 independent reflections
3381 reflections with *I* > 2σ(*I*)
*R*_int_ = 0.027

#### Refinement

*R*[*F* ^2^ > 2σ(*F* ^2^)] = 0.035
*wR*(*F* ^2^) = 0.100
*S* = 1.06
3789 reflections
193 parameters
H-atom parameters constrained
Δρ_max_ = 0.29 e Å^−3^
Δρ_min_ = −0.37 e Å^−3^

### 

Data collection: *APEX2* (Bruker, 2009 ▶); cell refinement: *SAINT* (Bruker, 2009 ▶); data reduction: *SAINT*; program(s) used to solve structure: *SHELXS97* (Sheldrick, 2008 ▶); program(s) used to refine structure: *SHELXL97* (Sheldrick, 2008 ▶); molecular graphics: *ORTEP-3* (Farrugia, 1997 ▶) and *DIAMOND* (Brandenburg, 1998 ▶); software used to prepare material for publication: *SHELXL97*.

## Supplementary Material

Crystal structure: contains datablocks global, I. DOI: 10.1107/S1600536810032228/ng5016sup1.cif

Structure factors: contains datablocks I. DOI: 10.1107/S1600536810032228/ng5016Isup2.hkl

Additional supplementary materials:  crystallographic information; 3D view; checkCIF report

## Figures and Tables

**Table 1:** Hydrogen-bond geometry (Å, °)

*D*—H⋯*A*	*D*—H	H⋯*A*	*D*⋯*A*	*D*—H⋯*A*
C13—H13⋯O2^i^	0.93	2.52	3.2548 (17)	136

Symmetry code: (i) 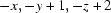.

**Fig. 1.**
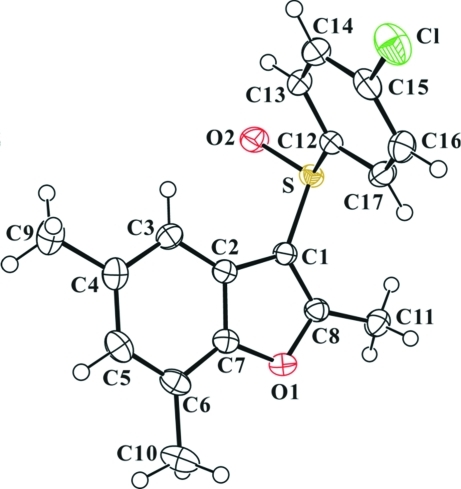
The molecular structure of the title compound with the atom numbering scheme. Displacement ellipsoids are drawn at the 50% probability level. H atoms are presented as a small spheres of arbitrary radius.

**Fig. 2.**
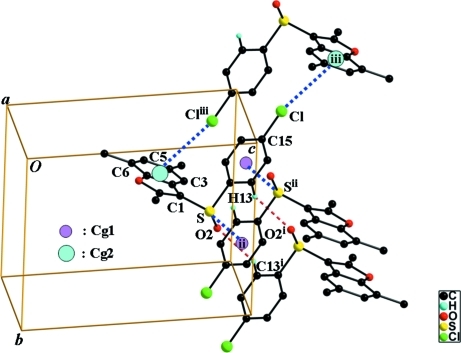
C—H···O, C—S···π, and C—Cl···π interactions (dotted lines) in the crystal structure of the title compound. Cg1 and Cg2 denote the ring centroids. [Symmetry codes: (i) -x, -y + 1, -z + 2; (ii) -x + 1, -y + 1 , -z + 2; (iii) -x + 1, -y, -z + 2.]

## supplementary crystallographic information

### Comment

A series of benzofuran ring system show remarkable pharmacological properties such as antimicrobial (Khan *et al.*, 2005), antifungal (Aslam *et al.*, 2006), antitumour and antiviral (Galal *et al.*, 2009) activities. These compounds widely occur in nature (Akgul & Anil, 2003; Soekamto *et al.*, 2003). As a part of our study of the substituent effect on the solid state structures of 3-(4-fluorophenylsulfinyl)-2-methyl-1-benzofuran analogues (Choi *et al.*, 2010*a*,*b*), we report the crystal structure of the title compound (Fig. 1).

The benzofuran unit is essentially planar, with a mean deviation of 0.005 (1) Å from the least-squares plane defined by the nine constituent atoms. The 4-chlorophenyl ring is nearly perpendicular to the benzofuran plane with a dihedral angle of 87.12 (3)° and is tilted slightly towards it. The molecular packing (Fig. 2) is stabilized by a weak intermolecular C—H···O hydrogen bond between the 4-chlorophenyl H atom and the oxygen of the S═O unit, with a C13—H13···O2^i^ (Table 1). The crystal packing (Fig. 2) is further stabilized by a weak intermolecular C—S···π interaction between the sulfur and 4-chlorophenyl ring of an adjacent molecule, with a C1—S···Cg1^ii^ [3.394 (2) Å] (Cg1 is the centroid of the C12–C17 4-chlorophenyl ring), and by an intermolecular C—Cl···π interaction between the chlorine and the benzene ring of a neighbouring benzofuran system, with a C15—Cl···Cg2^iii^ [3.800 (2) Å] (Cg2 is the centroid of the C2–C7 benzene ring).

### Experimental

77% 3-chloroperoxybenzoic acid (247 mg, 1.1 mmol) was added in small portions to a stirred solution of 3-(4-chlorophenylsulfanyl)-2,5,7-trimethyl-1-benzofuran (303 mg, 1.0 mmol) in dichloromethane (40 ml) at 273 K. After being stirred at room temperature for 4 h, the mixture was washed with saturated sodium bicarbonate solution and the organic layer was separated, dried over magnesium sulfate, filtered and concentrated at reduced pressure. The residue was purified by column chromatography (hexane–ethyl acetate, 1:1 v/v) to afford the title compound as a colourless solid [yield 79%, m.p. 419–420 K; *R*_f_ = 0.69 (hexane–ethyl acetate, 1:1 v/v)]. Single crystals suitable for X-ray diffraction were prepared by slow evaporation of a solution of the title compound in ethyl acetate at room temperature.

### Refinement

All H atoms were positioned geometrically and refined using a riding model, with C—H = 0.93 Å for aryl and 0.96 Å for methyl H atoms. *U*_iso_(H) = 1.2*U*_eq_(C) for aryl and 1.5*U*_eq_(C) for methyl H atoms.

### Crystal data

**Table:**

C_17_H_15_ClO_2_S	*Z* = 2
*M**_r_* = 318.80	*F*(000) = 332
Triclinic, *P*1	*D*_x_ = 1.390 Mg m^−^^3^
Hall symbol: -P 1	Mo *K*α radiation, λ = 0.71073 Å
*a* = 6.0790 (12) Å	Cell parameters from 8051 reflections
*b* = 10.2232 (19) Å	θ = 2.5–28.5°
*c* = 12.514 (2) Å	µ = 0.39 mm^−^^1^
α = 84.474 (9)°	*T* = 173 K
β = 80.121 (9)°	Block, colourless
γ = 85.991 (9)°	0.33 × 0.29 × 0.29 mm
*V* = 761.5 (3) Å^3^	

### Data collection

**Table:**

Bruker SMART APEXII CCD diffractometer	3789 independent reflections
Radiation source: rotating anode	3381 reflections with *I* > 2σ(*I*)
graphite multilayer	*R*_int_ = 0.027
Detector resolution: 10.0 pixels mm^-1^	θ_max_ = 28.5°, θ_min_ = 1.7°
φ and ω scans	*h* = −8→8
Absorption correction: multi-scan (*SADABS*; Bruker, 2009)	*k* = −13→13
*T*_min_ = 0.883, *T*_max_ = 0.897	*l* = −16→16
13813 measured reflections	

### Refinement

**Table:**

Refinement on *F*^2^	Primary atom site location: structure-invariant direct methods
Least-squares matrix: full	Secondary atom site location: difference Fourier map
*R*[*F*^2^ > 2σ(*F*^2^)] = 0.035	Hydrogen site location: difference Fourier map
*wR*(*F*^2^) = 0.100	H-atom parameters constrained
*S* = 1.06	*w* = 1/[σ^2^(*F*_o_^2^) + (0.0527*P*)^2^ + 0.253*P*] where *P* = (*F*_o_^2^ + 2*F*_c_^2^)/3
3789 reflections	(Δ/σ)_max_ = 0.001
193 parameters	Δρ_max_ = 0.29 e Å^−^^3^
0 restraints	Δρ_min_ = −0.37 e Å^−^^3^

### Special details

**Table:**

Geometry. All esds (except the esd in the dihedral angle between two l.s. planes) are estimated using the full covariance matrix. The cell esds are taken into account individually in the estimation of esds in distances, angles and torsion angles; correlations between esds in cell parameters are only used when they are defined by crystal symmetry. An approximate (isotropic) treatment of cell esds is used for estimating esds involving l.s. planes.
Refinement. Refinement of F^2^ against ALL reflections. The weighted R-factor wR and goodness of fit S are based on F^2^, conventional R-factors R are based on F, with F set to zero for negative F^2^. The threshold expression of F^2^ > 2sigma(F^2^) is used only for calculating R-factors(gt) etc. and is not relevant to the choice of reflections for refinement. R-factors based on F^2^ are statistically about twice as large as those based on F, and R- factors based on ALL data will be even larger.

### Fractional atomic coordinates and isotropic or equivalent isotropic displacement parameters (Å^2^)

**Table:**

	*x*	*y*	*z*	*U*_iso_*/*U*_eq_	
Cl	0.59001 (8)	0.01403 (4)	1.15921 (3)	0.04476 (12)	
S	0.39442 (5)	0.50713 (3)	0.84174 (2)	0.02515 (10)	
O1	0.70564 (15)	0.39917 (9)	0.55824 (7)	0.0275 (2)	
O2	0.14556 (16)	0.52738 (11)	0.85326 (8)	0.0345 (2)	
C1	0.4919 (2)	0.43493 (12)	0.71941 (10)	0.0238 (2)	
C2	0.3922 (2)	0.33367 (12)	0.67424 (10)	0.0233 (2)	
C3	0.2051 (2)	0.25904 (13)	0.70614 (11)	0.0268 (3)	
H3	0.1112	0.2693	0.7721	0.032*	
C4	0.1625 (2)	0.16917 (13)	0.63714 (12)	0.0298 (3)	
C5	0.3092 (3)	0.15433 (13)	0.53838 (12)	0.0323 (3)	
H5	0.2783	0.0928	0.4937	0.039*	
C6	0.4978 (2)	0.22683 (13)	0.50407 (10)	0.0299 (3)	
C7	0.5302 (2)	0.31615 (12)	0.57516 (10)	0.0249 (3)	
C8	0.6769 (2)	0.47050 (13)	0.64733 (10)	0.0255 (3)	
C9	−0.0407 (3)	0.08820 (16)	0.66938 (15)	0.0409 (4)	
H9A	−0.0003	0.0065	0.7076	0.061*	
H9B	−0.0967	0.0707	0.6052	0.061*	
H9C	−0.1544	0.1359	0.7157	0.061*	
C10	0.6554 (3)	0.21158 (16)	0.39876 (12)	0.0418 (4)	
H10A	0.6553	0.2929	0.3535	0.063*	
H10B	0.6078	0.1432	0.3619	0.063*	
H10C	0.8037	0.1888	0.4137	0.063*	
C11	0.8487 (2)	0.56565 (14)	0.64818 (12)	0.0322 (3)	
H11A	0.7992	0.6222	0.7056	0.048*	
H11B	0.8723	0.6177	0.5795	0.048*	
H11C	0.9862	0.5189	0.6600	0.048*	
C12	0.4487 (2)	0.36497 (13)	0.93215 (10)	0.0238 (2)	
C13	0.2739 (2)	0.31452 (14)	1.00741 (11)	0.0284 (3)	
H13	0.1297	0.3526	1.0104	0.034*	
C14	0.3172 (2)	0.20634 (15)	1.07826 (12)	0.0332 (3)	
H14	0.2020	0.1708	1.1290	0.040*	
C15	0.5339 (3)	0.15185 (14)	1.07258 (11)	0.0307 (3)	
C16	0.7104 (2)	0.20486 (15)	0.99957 (12)	0.0328 (3)	
H16	0.8551	0.1680	0.9976	0.039*	
C17	0.6671 (2)	0.31354 (14)	0.92966 (11)	0.0302 (3)	
H17	0.7835	0.3518	0.8814	0.036*	

### Atomic displacement parameters (Å^2^)

**Table:**

	*U*^11^	*U*^22^	*U*^33^	*U*^12^	*U*^13^	*U*^23^
Cl	0.0623 (3)	0.0352 (2)	0.0401 (2)	−0.00630 (17)	−0.02155 (19)	0.00593 (15)
S	0.02602 (17)	0.02658 (17)	0.02290 (16)	0.00133 (12)	−0.00348 (12)	−0.00547 (11)
O1	0.0279 (5)	0.0294 (5)	0.0231 (4)	−0.0013 (4)	0.0010 (3)	−0.0016 (3)
O2	0.0269 (5)	0.0442 (6)	0.0312 (5)	0.0108 (4)	−0.0041 (4)	−0.0072 (4)
C1	0.0238 (6)	0.0255 (6)	0.0217 (6)	0.0002 (5)	−0.0033 (4)	−0.0025 (4)
C2	0.0238 (6)	0.0231 (6)	0.0226 (6)	0.0028 (4)	−0.0047 (4)	−0.0018 (4)
C3	0.0236 (6)	0.0266 (6)	0.0292 (6)	0.0008 (5)	−0.0024 (5)	−0.0016 (5)
C4	0.0287 (6)	0.0242 (6)	0.0382 (7)	0.0010 (5)	−0.0121 (5)	−0.0008 (5)
C5	0.0425 (8)	0.0256 (6)	0.0319 (7)	0.0012 (5)	−0.0144 (6)	−0.0049 (5)
C6	0.0398 (7)	0.0266 (6)	0.0229 (6)	0.0054 (5)	−0.0071 (5)	−0.0028 (5)
C7	0.0270 (6)	0.0240 (6)	0.0231 (6)	0.0011 (5)	−0.0038 (5)	−0.0005 (4)
C8	0.0256 (6)	0.0272 (6)	0.0230 (6)	0.0010 (5)	−0.0040 (5)	−0.0011 (5)
C9	0.0332 (7)	0.0346 (8)	0.0575 (10)	−0.0058 (6)	−0.0121 (7)	−0.0059 (7)
C10	0.0617 (10)	0.0356 (8)	0.0258 (7)	0.0021 (7)	0.0004 (7)	−0.0076 (6)
C11	0.0278 (6)	0.0340 (7)	0.0341 (7)	−0.0053 (5)	−0.0042 (5)	0.0010 (5)
C12	0.0229 (6)	0.0281 (6)	0.0210 (6)	−0.0017 (5)	−0.0039 (4)	−0.0044 (5)
C13	0.0215 (6)	0.0352 (7)	0.0285 (6)	−0.0037 (5)	−0.0023 (5)	−0.0051 (5)
C14	0.0323 (7)	0.0382 (8)	0.0287 (7)	−0.0107 (6)	−0.0018 (5)	−0.0009 (6)
C15	0.0396 (7)	0.0290 (6)	0.0261 (6)	−0.0051 (5)	−0.0120 (5)	−0.0017 (5)
C16	0.0263 (6)	0.0370 (7)	0.0359 (7)	0.0023 (5)	−0.0091 (5)	−0.0028 (6)
C17	0.0229 (6)	0.0374 (7)	0.0286 (7)	−0.0006 (5)	−0.0006 (5)	−0.0009 (5)

### Geometric parameters (Å, °)

**Table:**

Cl—C15	1.7443 (15)	C9—H9A	0.9600
S—O2	1.4964 (10)	C9—H9B	0.9600
S—C1	1.7563 (13)	C9—H9C	0.9600
S—C12	1.8015 (13)	C10—H10A	0.9600
O1—C8	1.3700 (16)	C10—H10B	0.9600
O1—C7	1.3846 (16)	C10—H10C	0.9600
C1—C8	1.3627 (17)	C11—H11A	0.9600
C1—C2	1.4408 (17)	C11—H11B	0.9600
C2—C7	1.3920 (17)	C11—H11C	0.9600
C2—C3	1.3937 (18)	C12—C13	1.3863 (18)
C3—C4	1.3854 (19)	C12—C17	1.3900 (18)
C3—H3	0.9300	C13—C14	1.389 (2)
C4—C5	1.409 (2)	C13—H13	0.9300
C4—C9	1.511 (2)	C14—C15	1.387 (2)
C5—C6	1.392 (2)	C14—H14	0.9300
C5—H5	0.9300	C15—C16	1.389 (2)
C6—C7	1.3805 (18)	C16—C17	1.386 (2)
C6—C10	1.5048 (19)	C16—H16	0.9300
C8—C11	1.4780 (19)	C17—H17	0.9300
			
O2—S—C1	108.00 (6)	H9A—C9—H9C	109.5
O2—S—C12	106.40 (6)	H9B—C9—H9C	109.5
C1—S—C12	96.87 (6)	C6—C10—H10A	109.5
C8—O1—C7	106.14 (10)	C6—C10—H10B	109.5
C8—C1—C2	107.49 (11)	H10A—C10—H10B	109.5
C8—C1—S	124.61 (10)	C6—C10—H10C	109.5
C2—C1—S	127.86 (10)	H10A—C10—H10C	109.5
C7—C2—C3	119.63 (12)	H10B—C10—H10C	109.5
C7—C2—C1	104.61 (11)	C8—C11—H11A	109.5
C3—C2—C1	135.75 (12)	C8—C11—H11B	109.5
C4—C3—C2	118.34 (12)	H11A—C11—H11B	109.5
C4—C3—H3	120.8	C8—C11—H11C	109.5
C2—C3—H3	120.8	H11A—C11—H11C	109.5
C3—C4—C5	119.66 (13)	H11B—C11—H11C	109.5
C3—C4—C9	119.32 (13)	C13—C12—C17	121.40 (12)
C5—C4—C9	121.02 (13)	C13—C12—S	119.24 (10)
C6—C5—C4	123.56 (13)	C17—C12—S	119.23 (10)
C6—C5—H5	118.2	C12—C13—C14	119.03 (12)
C4—C5—H5	118.2	C12—C13—H13	120.5
C7—C6—C5	114.29 (12)	C14—C13—H13	120.5
C7—C6—C10	121.78 (14)	C15—C14—C13	119.40 (13)
C5—C6—C10	123.92 (13)	C15—C14—H14	120.3
C6—C7—O1	124.64 (12)	C13—C14—H14	120.3
C6—C7—C2	124.50 (12)	C14—C15—C16	121.63 (13)
O1—C7—C2	110.86 (11)	C14—C15—Cl	119.93 (11)
C1—C8—O1	110.89 (11)	C16—C15—Cl	118.45 (11)
C1—C8—C11	133.18 (12)	C17—C16—C15	118.88 (13)
O1—C8—C11	115.91 (11)	C17—C16—H16	120.6
C4—C9—H9A	109.5	C15—C16—H16	120.6
C4—C9—H9B	109.5	C16—C17—C12	119.56 (13)
H9A—C9—H9B	109.5	C16—C17—H17	120.2
C4—C9—H9C	109.5	C12—C17—H17	120.2
			
O2—S—C1—C8	137.22 (11)	C1—C2—C7—C6	179.56 (12)
C12—S—C1—C8	−113.01 (12)	C3—C2—C7—O1	179.84 (11)
O2—S—C1—C2	−40.20 (13)	C1—C2—C7—O1	0.32 (14)
C12—S—C1—C2	69.57 (12)	C2—C1—C8—O1	−0.20 (14)
C8—C1—C2—C7	−0.07 (14)	S—C1—C8—O1	−178.07 (9)
S—C1—C2—C7	177.71 (10)	C2—C1—C8—C11	−178.62 (14)
C8—C1—C2—C3	−179.47 (14)	S—C1—C8—C11	3.5 (2)
S—C1—C2—C3	−1.7 (2)	C7—O1—C8—C1	0.40 (14)
C7—C2—C3—C4	−0.10 (18)	C7—O1—C8—C11	179.11 (11)
C1—C2—C3—C4	179.23 (13)	O2—S—C12—C13	−11.33 (12)
C2—C3—C4—C5	0.85 (19)	C1—S—C12—C13	−122.43 (11)
C2—C3—C4—C9	−179.33 (12)	O2—S—C12—C17	172.79 (11)
C3—C4—C5—C6	−0.7 (2)	C1—S—C12—C17	61.69 (12)
C9—C4—C5—C6	179.49 (13)	C17—C12—C13—C14	−3.1 (2)
C4—C5—C6—C7	−0.2 (2)	S—C12—C13—C14	−178.86 (10)
C4—C5—C6—C10	179.96 (13)	C12—C13—C14—C15	0.4 (2)
C5—C6—C7—O1	−179.80 (12)	C13—C14—C15—C16	1.7 (2)
C10—C6—C7—O1	0.0 (2)	C13—C14—C15—Cl	−178.75 (11)
C5—C6—C7—C2	1.05 (19)	C14—C15—C16—C17	−1.1 (2)
C10—C6—C7—C2	−179.14 (13)	Cl—C15—C16—C17	179.36 (11)
C8—O1—C7—C6	−179.69 (12)	C15—C16—C17—C12	−1.6 (2)
C8—O1—C7—C2	−0.44 (14)	C13—C12—C17—C16	3.7 (2)
C3—C2—C7—C6	−0.92 (19)	S—C12—C17—C16	179.48 (11)

### Hydrogen-bond geometry (Å, °)

**Table:**

*D*—H···*A*	*D*—H	H···*A*	*D*···*A*	*D*—H···*A*
C13—H13···O2^i^	0.93	2.52	3.2548 (17)	136.

Symmetry codes: (i) −*x*, −*y*+1, −*z*+2.
